# Validation of 3-Space Wireless Inertial Measurement Units Using an Industrial Robot

**DOI:** 10.3390/s21206858

**Published:** 2021-10-15

**Authors:** Jaime Hislop, Mats Isaksson, John McCormick, Chris Hensman

**Affiliations:** 1 Department of Mechanical Engineering and Product Design Engineering, Swinburne University of Technology, Hawthorn, VIC 3122, Australia; misaksson@swin.edu.au; 2 Centre for Transformative Media Technologies, Swinburne University of Technology, Hawthorn, VIC 3122, Australia; jmccormick@swin.edu.au; 3 Department of Surgery, Monash University, Clayton, VIC 3168, Australia; chensman@lapsurg.net.au; 4 Department of Surgery, University of Adelaide, Adelaide, SA 5005, Australia; 5 LapSurgery Australia, Dandenong North, VIC 3175, Australia

**Keywords:** Inertial Measurement Unit (IMU), sensor fusion, sensor validation, Kalman filter, complementary filter, gradient descent filter

## Abstract

Inertial Measurement Units (IMUs) are beneficial for motion tracking as, in contrast to most optical motion capture systems, IMU systems do not require a dedicated lab. However, IMUs are affected by electromagnetic noise and may exhibit drift over time; it is therefore common practice to compare their performance to another system of high accuracy before use. The 3-Space IMUs have only been validated in two previous studies with limited testing protocols. This study utilized an IRB 2600 industrial robot to evaluate the performance of the IMUs for the three sensor fusion methods provided in the 3-Space software. Testing consisted of programmed motion sequences including 360° rotations and linear translations of 800 mm in opposite directions for each axis at three different velocities, as well as static trials. The magnetometer was disabled to assess the accuracy of the IMUs in an environment containing electromagnetic noise. The Root-Mean-Square Error (RMSE) of the sensor orientation ranged between 0.2° and 12.5° across trials; average drift was 0.4°. The performance of the three filters was determined to be comparable. This study demonstrates that the 3-Space sensors may be utilized in an environment containing metal or electromagnetic noise with a RMSE below 10° in most cases.

## 1. Introduction

Motion capture has important and evolving applications in research, sports science, clinical assessment, and rehabilitation, as well as in animation and virtual reality. Optical Motion Capture Systems (OMCSs) are considered the gold standard due to their sub-millimetre accuracy; however, OMCSs depend on multiple infrared cameras that require line of sight to the person or object of interest. Hence, Optical Motion Capture (OMC) is expensive and is often performed in a dedicated indoor lab, which makes data collection in a natural or dynamic setting prohibitive [1].

An Inertial Measurement Unit (IMU) uses a combination of accelerometers, gyroscopes, and magnetometers to obtain orientation data. IMUs are becoming more ubiquitous for motion tracking due to the ability to employ them in a wider range of environments; however, it is still common practice to benchmark their performance against another highly accurate system prior to experimental use [2]. The 3-Space IMUs validated in this paper are relatively new and have not been commonly utilized. This system can either be used with the provided motion capture software, or data can be logged from individual IMUs using custom-written scripts.

Different 3-Space IMU models have been utilized in studies implementing real-time posture feedback systems. Abyarjoo et al. [3] and Yan et al. [4] presented systems which used different 3-Space modules to alert the user to deviations in posture from a previously calibrated ideal position. Both studies described the implementation of their systems, although these studies they did not present any preliminary data analyzing the corrective impact it had on posture.

Williams et al. [5] performed a validation study of one 3-Space IMU by incrementally tilting the IMU between 0° and 90° about each axis, verified by a goniometer. This process was repeated 10 times. Error scores calculated between the IMU and the goniometer showed a maximum typical error of 0.03° in any of the three planes. The small typical error in this study was promising; however, measurements were only taken from one IMU when static over a limited range of orientations.

Ligorio et al. [6] used the 3-Space IMUs in combination with the Vicon OMCS to compare the accuracy of functional calibration techniques applied to each motion tracking system. The functional calibration method consisted of arm rotations, as well as flexion and extension of the elbow and a static standing pose. This is an extension of the traditional anatomical calibration method of each system, where a short recording of the participant in a static pose is used to compose or adjust a biomechanical model. This data was applied to initialize the position of a representative model of the elbow. The Root-Mean-Square Error (RMSE) between systems during subsequent movement of the upper extremities was approximately 4°.

Variables relating to the comprehensiveness of an IMU validation study include the number of participants and the range of anthropometry between them, trial duration, the number of joints analyzed, and the nature of the motions performed by participants [2]. Sources of discrepancy arise from differences in sensor placement and the conventions of the modeling software used in conjunction with each system [2]. Additionally, with respect to the laboratory or capture volume, considerations include magnetic interference, especially as it differs throughout a space used in data collection, and the alignment of the global reference frames between the systems [7]. As a result, it is difficult to determine whether the disagreement between the systems is related to hardware, software, or study design factors. Directly comparing IMU orientation to another system of high accuracy without the added complexity of biomechanical modeling and the possible error introduced by the magnetometer is a beneficial validation method to precede the traditional process of validation with an OMCS during human motion trials.

The purpose of this study was to evaluate the performance of the 3-Space IMUs and determine the accuracy of the sensor fusion filters provided in 3-Space software without the use of the magnetometer. The IMU magnetometers were disabled throughout testing as these sensors will be used to investigate the ergonomics of surgeons in operating theaters which contain significant electromagnetic noise. It is essential to have measurement technology that is not affected by this. This is the most thorough validation process performed using these IMUs to date; this protocol can also be applied to other IMUs and provides a testing benchmark for comparison.

## 2. Materials and Methods

### 2.1. Measurement Setup

**Figure 1:**
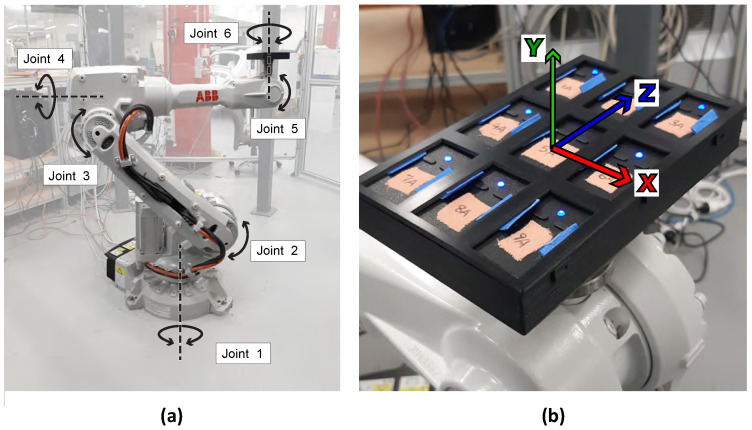
(**a**) IRB 2600 industrial robot, with all joints and axes of rotation labelled (**b**) IMUs positioned in a custom-made holder attached to the robot mounting interface, with the default coordinate system of IMUs superimposed on the image.

The performance of individual 3-Space Wireless 2.4 GHz DSSS IMUs (Yost Labs, Portsmouth, OH, USA) was tested using an IRB 2600 industrial robot (ABB, Zürich, Switzerland), shown in Figure 1a. The IRB 2600 robot from ABB Robotics was used to provide a reference for validation as it allowed an identical programmed path to be repeated at multiple velocities to test the performance of the IMUs under dynamic conditions. By executing rotation and translation motion sequences for one axis at a time, the level of precision for each of the IMU axes could be calculated. Nine IMUs were attached to the robot in a grid using the 3D printed mount shown in Figure 1b to ensure that each of the sensors had the same orientation while the robot moved the IMUs along the programmed paths. IMU orientation was calculated from raw accelerometer, gyroscope, and magnetometer values using one of the sensor fusion methods available through the 3-Space Mocap Studio program: the gradient descent filter (QGrad), the complementary filter (QComp), or the Kalman filter. The QGrad filter iteratively optimizes an estimated orientation based on previous states and raw data using gradient descent or differentiation methods to determine the local minimum of the squared error function [8]. The QComp filter applies a low-pass filter to accelerometer data, as accelerometers are susceptible to short-term changes due external forces, and a high-pass filter is applied to gyroscope data, as gyroscope measurements are susceptible to drift over long periods of time. These measurements are then combined to produce orientation [9]. Kalman filters use previous measurements combined with incoming raw data to correct and optimize the output orientation with linear regression [10]. Each sensor fusion method was evaluated with the magnetometer disabled to assess their performance in an environment containing electromagnetic noise. Data was also collected using the Kalman filter with the magnetometer enabled for comparison. Without the magnetometer, the gyroscope only provides a relative heading in the horizontal plane [11], which is combined with a known starting position determined during calibration to calculate the orientation with respect to the global coordinate system. The sample rate was set to be 248.0159 Hz as this is the logging frequency used by the IRB 2600. IMU settings were the same for all sensors. The accelerometer trust values, which dictate how reliable the accelerometer values are and how they contribute to the output orientation, were identical for all sensors although varied depending on the selected filter. These settings are given in Table 1.

### 2.2. Measurement Protocol

Rotational, translational, and stationary motion sequences were performed. Firstly, 360° revolutions were performed by the robot arm in parallel to the X, Y, and Z axes shown in Figure 1b in both positive and negative directions, thereby studying the change in one IMU axis at a time. The programmed angular velocities about each axis were 45°/s, 90°/s, and 360°/s. These angular velocities were chosen to provide a comprehensive validation of the 3-Space sensors. However, for our future work of tracking the motion of surgeons while operating the velocity of their upper body movements will be between 0°/s and 45°/s [12,13]. After the initial acceleration, the robot follows the programmed path at the defined speed. The acceleration is optimized to use all available motor torque, unless a reduction of acceleration is ordered.

To simplify the logging of the IRB 2600 orientation, only one robot joint was rotated during each motion sequence. The motor angles of each joint of the robot were logged with ABB TuneMaster at a sample rate of 248.0159 Hz. This was used to derive a reference signal to calculate the accuracy of the IMU orientation, described in more detail below. For the rotational motion sequences, robot joints one, two, three, and five were kept fixed whereas the other joints were programmed according to Table 2.

To determine if the measured orientation of the IMUs remained constant during linear motion, despite the signal artifacts created by this movement, a distance of 800 mm was traveled along each axis shown in Figure 1b, followed by a motion of 800 mm in the opposite direction back to its original position at an initial speed of 250 mm/s. The velocity was increased after each set of repetitions to 500 mm/s and then 1000 mm/s. During translation, the orientation and coordination of the individual joints of the robot was more complex, although the reference orientation was constant. This motion is described in Table 2, using the distance of the tool center point (TCP) from the point of origin defined by the IRB 2600.

Following this, the plate was kept stationary to assess variance in IMU readings while static. This was done in three different orientations, with the sensor mount rotated so that each of the local sensor axes depicted in Figure 1b was pointing directly upwards for 30 s at a time. These positions were achieved by programming the motions of joints four and six, as described in Table 2. Transitions between positions were executed in less than 0.3 s.

Additionally, to examine drift, the data was logged from the IMUs in a single orientation over a 1-h period. This test was conducted with the Y-axis of the IMUs aligned with gravity, as described in Table 2, at a sampling rate of 20 Hz. The Kalman filter with the magnetometer disabled was used for sensor fusion.

### 2.3. Data Analysis

Following data collection, MATLAB (The Mathworks, Inc., Natick, MA, USA) was used to analyze the signals.

The IMU data was exported from the Mocap Studio as a Biovision Hierarchy (BVH) file in Euler angles with a ZYX rotation sequence, which were converted to quaternions in MATLAB. A quaternion defined by $q=(q_{1},q_{2},q_{3},q_{4})$ is a four-dimensional representation of a rotation in three-dimensional space. The quaternion relates to rotation (θ) around a normalized vector $n=[n_{1},n_{2},n_{3}]$ according to:

(1) $$\begin{array}{cc} q_{1} & =\cos\left(\frac{\theta }{2}\right) \end{array}$$

(2) $$\begin{array}{cc} q_{2} & =n_{1}\times \sin\left(\frac{\theta }{2}\right) \end{array}$$

(3) $$\begin{array}{cc} q_{3} & =n_{2}\times \sin\left(\frac{\theta }{2}\right) \end{array}$$

(4) $$\begin{array}{cc} q_{4} & =n_{3}\times \sin\left(\frac{\theta }{2}\right) \end{array}$$

where $n_{1}^{2}+n_{2}^{2}+n_{3}^{2}=1$. The difference between the reference quaternion $q_{r}$ and a measured quaternion $q_{m}$ is calculated by

(5) $$q_{d}=q_{m}\times q_{r}^{\prime }=\left(q_{d1},q_{d2},q_{d3},q_{d4}\right)$$

where $q^{\prime }$ represents the conjugate or inverse of q which is $q^{\prime }=\left(q_{1},-q_{2},-q_{3},-q_{4}\right)$. Quaternion multiplication was performed as described in [14]. The 3-D orientation of each series was adjusted with reference to the first frame of the data by multiplying the signal by the inverse quaternion of the first frame using Equation (5). As $\left(q_{1},q_{2},q_{3},q_{4}\right)=\left(-q_{1},-q_{2},-q_{3},-q_{4}\right)$, any discontinuities in the signal were corrected with a simple logic function where each term of the signal was multiplied by −1 from the disconnected frame onwards. This did not change the orientation being represented; however, without this correction subsequent error analysis would be impacted.

The motor angles of the IRB 2600 were logged to obtain the reference signal for the orientation of the sensors. The arm angles of joints one, two, three, and four were calculated from the corresponding motor angles by utilizing the gear ratio of each joint. The arm angle calculations for joints five and six required a transformation function to address the coupling present between joints four, five, and six. Then the angle of each joint was combined using rotation matrices to obtain the orientation of the TCP. This orientation was converted to a quaternion.

The data from the IMUs and measurements logged from the robot were aligned using a correlation function. Following this, the error between the measured IMU signals and the IRB 2600 signal was calculated. This was done by finding the difference between the measured and reference signals as a quaternion rotation using Equation (5). The quaternion error was then converted to the axis-angle convention:

(6) $$\begin{array}{cc} \theta_{d} & =2\arccos\left(q_{d1}\right) \end{array}$$

(7) $$\begin{array}{cc} n_{d1} & =\frac{q_{d2}}{\sin\left(\arccos\left(q_{d1}\right)\right)} \end{array}$$

(8) $$\begin{array}{cc} n_{d2} & =\frac{q_{d3}}{\sin\left(\arccos\left(q_{d1}\right)\right)} \end{array}$$

(9) $$\begin{array}{cc} n_{d3} & =\frac{q_{d4}}{\sin\left(\arccos\left(q_{d1}\right)\right)} \end{array}$$

which can be derived from Equations (1)–(5). The value $\theta_{d}$ describes the angular difference between the reference and measured orientations. This was used to calculate the RMSE across all nine sensors for each filter method and motion sequence.

Drift was calculated using linear regression to determine the gradient of the IMU angles recorded over the 1-h time period. The difference in the mean orientation between the last five seconds and the first five seconds was also evaluated.

Significant differences in IMU orientation related to filter performance, velocity, and sensor differences were analyzed in MATLAB using one-way Analysis of Variance (ANOVA). A *p*-value less than 0.05 was considered statistically significant. If the ANOVA test produced a significant result, a pairwise comparison was also completed using a multiple comparison test.

## 3. Results

**Figure 2:**
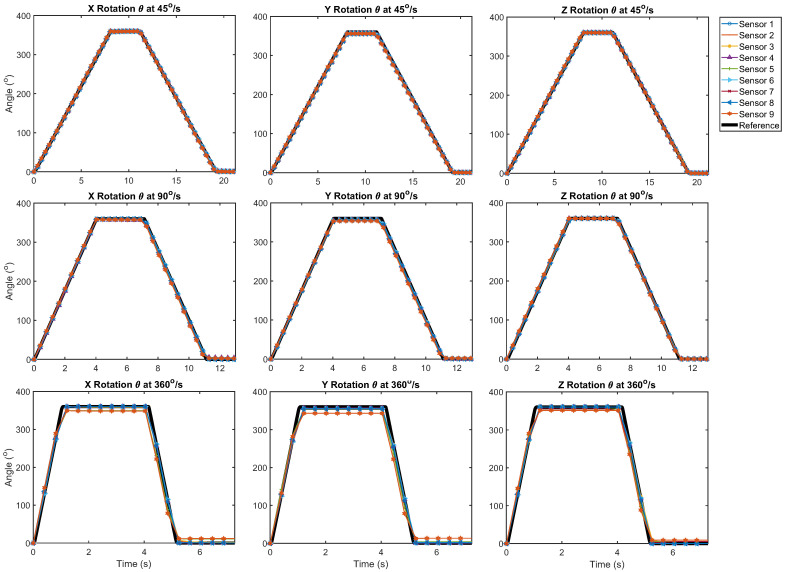
Rotation θ output of the individual IMUs fused with the Kalman filter for all angular velocities, stratified by axis of rotation. Derived from (1), $\theta =2\arccos\left(q_{1}\right)$.

**Figure 3:**
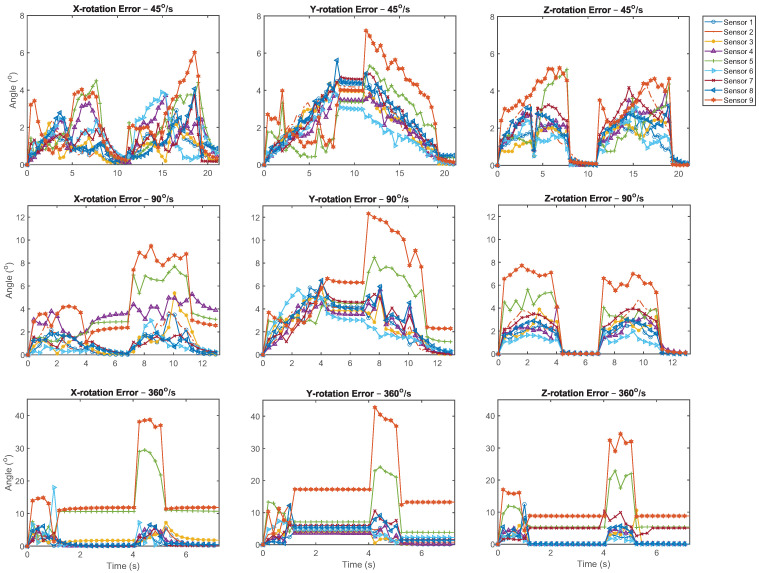
Rotation error of the individual IMUs fused with the Kalman filter without the magnetometer, stratified by axis of rotation and angular velocity.

Without the magnetometer enabled, the mean error of the sensors throughout the trials ranged between 0.24° and 12.5° as summarized in Table 3. The error ranged from 2.90° to 100° when the magnetometer was enabled. Figure 2 shows that the IMUs provided a reasonable approximation for the orientation of the sensor mount throughout the rotational motion sequence. However, in Figure 3 it can be seen that there were differences in the reactivity of the IMUs at each time point. Additionally, the sensors did not always accurately detect the 360° range of motion performed in the motion sequence. During the rotation sequence, the performance of the three filters without the magnetometer was comparable in all but one instance, as the difference between the average sensor error for each fusion method was not statistically significant. This analysis is detailed in Table 4. Increased error was observed for the rotation about the Y-axis in comparison to the other axes at 45°/s for all filters without the magnetometer, as detailed in Table 5, although this was not seen at higher velocities.

Interestingly, the Kalman filter with the magnetometer enabled performed equally as well as the other filters that were used without the magnetometer when the angular velocity was 360°/s for all axes. The QGrad and QComp filters demonstrated a significant increase in error when the angular velocity increased. Conversely, the error of the Kalman filter with the magnetometer enabled decreased when the angular velocity decreased, as detailed in Table 6.

**Figure 4:**
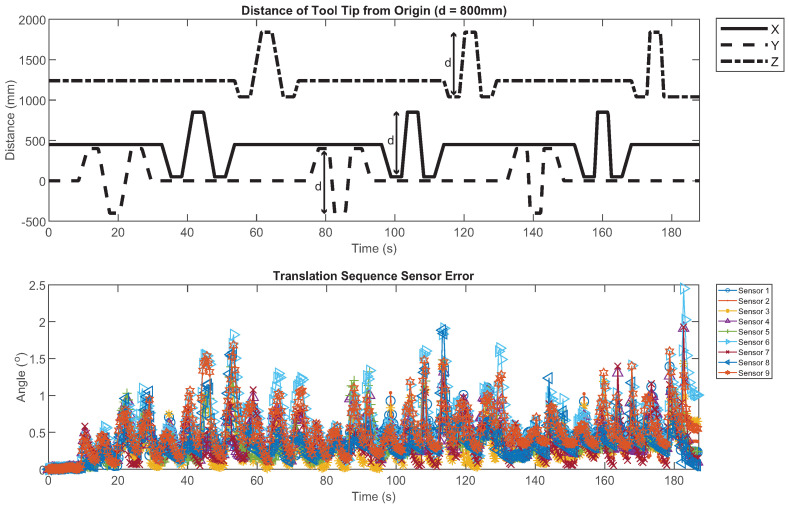
The programmed position of the tool center point (TCP) in mm from the point of origin defined by the IRB 2600, using the local coordinate system of the IRB 2600 (**top**). Translation error of the individual IMUs fused with the Kalman filter without the magnetometer (**bottom**).

Lower RMSE values were observed during the translation sequence compared to the other tests, as shown in Table 3. The results of the three filters without the magnetometer were significantly different during the translation sequence when compared using ANOVA, although all errors were less than 1°. As shown in Figure 4, 99% of the errors in IMU output were less than 1.5°; the maximum observed error was 2.5°.

**Figure 5:**
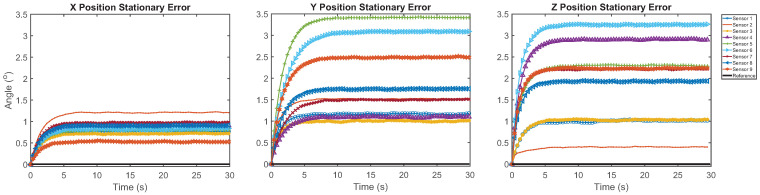
Stationary error of the individual IMUs fused with the Kalman filter without the magnetometer plotted alongside reference. The three positions indicate the IMU sensor axis aligned with gravity.

During the 30-s stationary tests, the error of the sensor fusion methods without the magnetometer ranged between 0.82 ± 0.18° and 2.73 ± 2.83° as stated in Table 3. Figure 5 shows that there was a settling time which occurred during the first five seconds of the test; the signal noise was minimal after this time. When the error is calculated without the initial 5 s of the signal, the RMSE was between 0.03 ± 0.01° and 0.12 ± 0.09°. There were significant differences between the filters depending on which axis was aligned with gravity. The Kalman filter with the magnetometer enabled performed equally as well as the other filters in the Y position. This was the first position in the programmed sequence and a one-point calibration was performed with the IMUs in this orientation immediately prior to data collection, which explains this result.

**Figure 6:**
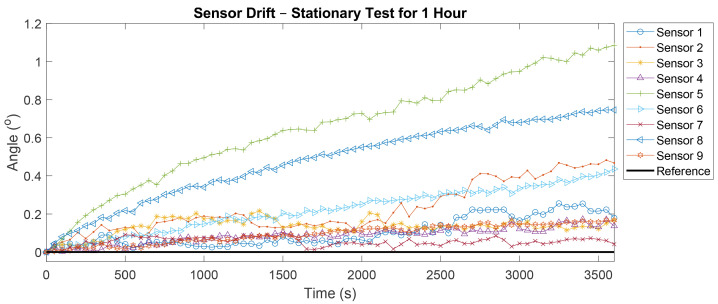
Sensor drift of the individual IMUs while stationary over a one-hour period fused with the Kalman filter without the magnetometer plotted alongside reference.

During the one-hour test, IMU drift was observed ranging between 0.04° and 1.08°, as shown in Figure 6. The average drift calculated using linear regression and difference in means was 0.43 ± 0.36° and 0.37 ± 0.32°, respectively.

Statistically significant differences were observed between the performance of individual sensors, although the sensors which showed the largest error changed with the selected filter.

IMUs demonstrating the largest RMSE differed between static and dynamic trials. This can be observed between Figure 3 and Figure 6. It is likely that the cause of the error was different between these trials. During rotation, the cause of error may have primarily been due to accelerometer or gyroscope performance as well as IMU reactivity. Whereas during the static trials, drift would most likely be associated with filter accuracy.

## 4. Discussion

The measured RMSE of the sensors ranged from 0.24° to 12.5° for all of the filters when the magnetometers were disabled. This was significantly higher than the typical error of 0.03° reported by Williams et al. [5]; however, that study only compared 300 data points in total. In comparison, our study logged data continuously from nine sensors at a frequency of 248.0159 Hz during 360° rotations and 800 mm translations in positive and negative directions for each axis at varying speed, as well as stationary tests in multiple positions to measure noise and drift. Measurements in the study by Williams et al. were only taken while the IMU was static, whereas our study examined the effects of dynamic movement on IMU orientation accuracy and demonstrated a positive correlation between angular velocity and error. Additionally, the one-hour stationary test to assess drift was longer than the trials conducted by Williams et al. [5] or Ligorio et al. [6] and produced results within the manufacturer specifications of 2.5°/h [15].

A significant difference in filter performance was not observed during the rotation sequences. There were significant differences between fusion methods for the translational and stationary tests. For the 30-s stationary test, the result may have been impacted by the alignment process and differences in settling time of the sensors; minimal sensor noise was observed after this initial settling time. According to the manufacturer, the Kalman filter is the most computationally expensive. The performance of the QGrad and QComp filters are stated as being comparable to the Kalman filter [15]. This aligns with the results of this study.

Unlike the other filters, the accuracy of the Kalman filter with the magnetometer enabled significantly improved as the velocity increased during the rotational motion sequence. This may be explained by the use of variable trust values to determine the contribution of the accelerometer and magnetometer values to the final result depending on the velocity being measured by the gyroscope [15]. This means the magnetometer and accelerometer are trusted more when a sensor is static or moving at a lower velocity than when it is moving at a higher velocity. The decreased trust in the magnetometer, which was significantly impacted by the electromagnetic noise in the surrounding environment, would have likely accounted for the reduction in error as the angular velocity increased. It should be noted that trust values differ between the filters. Sensor settings may also be changed to use static trust values [15].

There are many commercially available IMU systems; one of the most common systems for biomechanical analysis is the Xsens which has been validated and used in several studies [2,16]. Other commercial systems include the Perception Neuron [17], SmartSuit Pro from Rokoko [18], and GaitSmart [19]. Available systems range from off-the-shelf solutions with a fixed configuration, to individual, highly customizable IMUs which give the user access to the raw data. A common method of validating IMU sensors is to have participants wear them along with reflective markers and have their performance compared to an OMCS while the participant performs basic movements [2,6,16,17]. This is valuable to determine the suitability of the IMU system for biomechanical applications. However, this protocol introduces several layers of complexity. Firstly, differences in convention may exist between the systems related to the definition of local coordinate systems and joint centers of rotation [2,20]. Secondly, the biomechanical models used by the systems may have different spatial and rotational offsets to each other [2]. Thirdly, because the IMU sensors and reflective markers are placed in different locations on the skin, the motions tracked by the two systems are impacted in disparate ways by soft-tissue artifacts [2,20]. This means that the measured error is impacted by factors relating to the software, physical setup of the systems, and the hardware. The current study utilized the IRB 2600 robot for validation to remove these confounding variables.

The design of the testing sequences to assess rotational and translational error, as well as stationary drift, separately for each axis is considered a strength of this study. The repeatability of this method means that it would be a suitable way to compare various IMU systems. It is recognized that in other environments enabling the magnetometers would likely improve the accuracy of the IMU orientation in the horizontal plane, which corresponded with the rotation about the sensor’s Y-axis in the current study. However, quantifying the performance of these sensors in an environment with electromagnetic noise is considered a valuable contribution of this study, as it evaluates the usability of IMUs in similar settings.

## 5. Conclusions

In conclusion, this study examined the accuracy of the 3-Space IMU sensors and provides a possible method of comparing other IMU systems in the future with high repeatability. Rotational accuracy varied with velocity; the RMSE fluctuated between 1.7° and 12.5° with the magnetometer disabled. Minimal error (RMSE < 3°) was observed in the signal during translation and while the sensors were stationary. The performance of the filters available in the provided software was comparable without the use of the magnetometer. Performing this validation study with the magnetometer disabled provides an evaluation of the usability of these IMU sensors for use in environments with electromagnetic noise. Further work remains to determine the accuracy of the 3-space sensors during more complex motion sequences and human movement tracking. Following this, it is the intention of our research group to use this sensor system for joint angle analysis in the context of investigating the ergonomics of surgeons performing traditional laparoscopic and robot-assisted laparoscopic surgery.

## Figures and Tables

**Table 1:** Accelerometer trust values.

Filter	Static Trust Value	Confidence Range	
		Minimum	Maximum
QComp	—	0.00001	0.010
QGrad	0.057	—	—
Kalman	—	0.001	0.063

**Table 2:** Starting position and motion sequences for the IRB 2600 robot. Starting position is provided in joint coordinates for all six joints (J1, J2, J3, etc.). For rotations, the motion is described by the rotation of the individual joints. For translations, the motion is described by the location of the tool center point (TCP) in mm in a work-object coordinate system which is rotated 45° around the vertical Z-axis compared to the robot’s default base coordinate system. The TCP is a projected point 103 mm away from the robot mounting plate in a direction normal to the robot mounting interface.

Motion Sequence	Axis in Local Sensor Coordinate System	Axis in Work-Object Coordinate System	Starting Position	Motion
Rotation	X	X	J1: 45°, J2: —45°, J3: 45°,	J4: 0°→ —360°, —360°→ 0°
			J4: 0°, J5: —90°, J6: —90°	
	Y	Z	J1: 45°, J2: —45°, J3: 45°,	J6: —90°→ 270°, 270°→ —90°
			J4: 0°, J5: —90°, J6: —90°	
	Z	Y	J1: 45°, J2: —45°, J3: 45°,	J4: 0°→ —360°, —360°→ 0°
			J4: 0°, J5: —90°, J6: 0°	
Translation	X	X	J1: 45°, J2: —45°, J3: 45°,	TCP: (450, 0, 1240) → (50, 0, 1240),
			J4: 0°, J5: —90°, J6: —90°	(50, 0, 1240) → (850, 0, 1240),
				(850, 0, 1240) → (50, 0, 1240),
				(50, 0, 1240) → (450, 0, 1240)
	Y	Z	J1: 45°, J2: —45°, J3: 45°,	TCP: (450, 0, 1240) → (450, 0, 1040),
			J4: 0°, J5: —90°, J6: —90°	(450, 0, 1040) → (450, 0, 1840),
				(450, 0, 1840) → (450, 0, 1040),
				(450, 0, 1040) → (450, 0, 1240)
	Z	Y	J1: 45°, J2: —45°, J3: 45°,	TCP: (450, 0, 1240) → (450, 400, 1240),
			J4: 0°, J5: —90°, J6: —90°	(450, 400, 1240) → (450, —400, 1240),
				(450, —400, 1240) → (450, 400, 1240),
				(450, 400, 1240) → (450, 0, 1240)
Stationary	X	X	J1: 45°, J2: —45°, J3: 45°,	—
(30-s test)			J4: —90°, J5: —90°, J6: 180°	
	Y	Z	J1: 45°, J2: —45°, J3: 45°,	—
			J4: 0°, J5: —90°, J6: 0°	
	Z	Y	J1: 45°, J2: —45°, J3: 45°,	—
			J4: —90°, J5: —90°, J6: 90°	
Stationary	Y	Z	J1: 45°, J2: —45°, J3: 45°,	—
(1-h test)			J4: 0°, J5: —90°, J6: 0°	

**Table 3:** RMSE of the angular difference between measured and reference values for each IMU data fusion method during rotation, translation, and stationary sequences. Axis refers to the IMU axis of rotation for the rotation sequence and the IMU axis aligned with gravity for the stationary trial.

Axis	Speed	QGrad without mag.	QComp without mag.	Kalman without mag.	Kalman with mag.
		RMSE (°)	RMSE (°)	RMSE (°)	RMSE (°)
**Rotation sequence**					
X	45°/s	2.46 ± 1.62	2.76 ± 0.48	1.69 ± 0.56	44.13 ± 23.27
	90°/s	4.01 ± 3.51	3.94 ± 1.50	2.30 ± 1.63	26.97 ± 17.54
	360°/s	10.70 ± 11.02	9.51 ± 5.10	5.20 ± 5.92	9.83 ± 6.44
Y	45°/s	4.55 ± 1.45	3.99 ± 0.92	2.80 ± 0.47	47.28 ± 32.48
	90°/s	5.14 ± 3.16	6.02 ± 1.63	3.75 ± 1.31	31.71 ± 29.89
	360°/s	10.41 ± 7.62	9.11 ± 4.25	6.55 ± 5.55	7.05 ± 4.58
Z	45°/s	2.75 ± 1.49	2.64 ± 0.45	2.13 ± 0.57	38.81 ± 23.27
	90°/s	4.90 ± 4.16	3.68 ± 1.20	2.35 ± 1.18	28.10 ± 18.98
	360°/s	12.5 ± 12.23	9.52 ± 4.89	4.92 ± 4.68	10.68 ± 7.02
**Translation sequence**	—	0.24 ± 0.04	0.83 ± 0.05	0.48 ± 0.10	7.11 ± 4.10
**Stationary (30-s test)**					
X	—	2.67 ± 1.96	1.27 ± 0.58	0.82 ± 0.18	34.51 ± 28.65
Y	—	2.73 ± 2.83	1.33 ± 0.59	1.81 ± 0.84	2.88 ± 3.50
Z	—	1.78 ± 0.98	3.78 ± 1.87	1.86 ± 0.91	100 ± 57.84

**Table 4:** Results of ANOVA and multiple comparison tests for the difference in mean error for QComp, QGrad, and Kalman filters. Axis refers to the IMU axis of rotation for the rotation sequence and the IMU axis aligned with gravity for the stationary trial. Significant differences (*p* < 0.05) indicated with an asterisk (*). Multiple comparison test results are not included for the Kalman filter with the magnetometer enabled because it is the instances of comparable performance that are of interest in this case.

Axis	Speed	ANOVA *p*-Value (without mag.)	Multiple Comparison Test *p*-Value			ANOVA *p*-Value (with mag.)
			QGrad v QComp	QGrad v Kalman	QComp v Kalman	
**Rotation sequence**						
X	45°/s	0.0928	—	—	—	<0.0001 *
	90°/s	0.2483	—	—	—	<0.0001 *
	360°/s	0.308	—	—	—	0.4187
Y	45°/s	0.0044 *	0.4906	0.0037 *	0.0532	<0.0001 *
	90°/s	0.1064	—	—	—	<0.0001 *
	360°/s	0.3918	—	—	—	0.4456
Z	45°/s	0.3498	—	—	—	<0.0001 *
	90°/s	0.1359	—	—	—	<0.0001 *
	360°/s	0.1535	—	—	—	0.2219
**Translation sequence**	—	<0.0001 *	<0.0001 *	<0.0001 *	<0.0001 *	<0.0001 *
**Stationary (30-s test)**						
X	—	0.008 *	0.0499 *	0.008 *	0.7005	<0.0001 *
Y	—	0.2422	—	—	—	0.4365
Z	—	0.0052 *	0.0101 *	0.9908	0.0137 *	<0.0001 *

**Table 5:** Results of ANOVA and multiple comparison tests for the difference in mean error about X, Y, and Z axes at 45°/s for each filter without the magnetometer. Significant differences (*p* < 0.05) indicated with an asterisk (*).

Filter	Speed	ANOVA *p*-Value	Multiple Comparison Test *p*-Value		
			X v Y	X v Z	Y v Z
QGrad	45°/s	0.0156 *	0.0201 *	0.9138	0.049 *
QComp	45°/s	0.0003 *	0.0015 *	0.9115	0.0005 *
Kalman	45°/s	0.007 *	0.0005 *	0.2082	0.0347 *

**Table 6:** Results of ANOVA and multiple comparison tests for the difference in mean error during rotations executed at 45°/s, 90°/s, and 360°/s. Significant differences (*p* < 0.05) indicated with an asterisk (*).

Filter	Speed	ANOVA *p*-Value	Multiple Comparison Test *p*-Value		
			45°/s v 90°/s	45°/s v 360°/s	90°/s v 360°/s
Qgrad	X	0.0372 *	0.8775	0.0411 *	0.1107
	Y	0.0317 *	0.9636	0.0429 *	0.0736
	Z	0.027 *	0.8182	0.0282 *	0.0998
Qcomp	X	0.0002 *	0.6999	0.0003 *	0.0022
	Y	0.0018 *	0.2625	0.0013 *	0.0555
	Z	<0.0001 *	0.734	0.0001 *	0.0008 *
Kalman nomag	X	0.1026	—	—	—
	Y	0.0614	—	—	—
	Z	0.0834	—	—	—
Kalman mag	X	0.0013 *	0.1083	0.0008 *	0.1088
	Y	0.0098 *	0.4149	0.0076 *	0.124
	Z	0.0093 *	0.4219	0.0072 *	0.1165

## Data Availability

The data presented in this study are openly available in FigShare at https://doi.org/10.6084/m9.figshare.16817308.v1.
